# The Treatment of Bacteriuria

**Published:** 1907-11-09

**Authors:** 


### Points in Treatment.

### THE TREATMENT OF BACTERIURIA.

Bacteriuria may be treated in two main ways, namely (1) by internal medication, (2) by antiseptic lavage of the bladder. These two methods may be used simultaneously; in severe cases of cystitis the latter is undoubtedly the more efficacious of the two, but it is with the former that we propose to deal in the present article. Lavage of the bladder is a measure which is not resorted to, especially in private practice, if it can be done without; not only is it disagreeable to the patient, but it is also a nuisance to the busy doctor, for it takes up a great deal of time, especially if it has to be repeated daily or more than once a day over a long period.

It is of interest, therefore, to know exactly what are the limitations of internal medication in the cure of pyuria.

### Internal Medication.

In the first place it is obvious that one must rely almost entirely upon internal medication if the source of the micro-organisms is the kidney. It therefore becomes necessary to use every possible means of determining the origin of the bacteriuria. The patient's history may suggest its renal origin, and the symptoms and signs may confirm this; on the other hand, this may not be so, and reliance must be placed upon the characters of the urine. Pus in an acid urine is in favour of suppuration in one kidney unless the amount of pus is very small; pus in an alkaline urine suggests either cystitis, or suppuration in both kidneys. The reason why pus in an acid urine suggests that there is a suppurative lesion in one kidney is, of course, that the pus itself should make the urine slightly alkaline, so that when the urine is still acid, notwithstanding the presence of pus in quantity, the purulent alkaline urine from one kidney must be mixing with the healthy acid urine from the other, the result being an acid urine containing pus. If there is more albumen present than can be accounted for by the pus alone—and pus accounts for comparatively little albumen—the argument is in favour of renal trouble. Microscopical examination of the centrifugalised deposit may discover renal cells, tube casts, or the typical epithelium from the pelvis of the kidney, showing that there is renal mischief whether there is cystitis at the same time or not.

### Its Results.

The question is, however, "To what extent can I cure bacteriuria by internal medication?" The answer to this question has hitherto been based largely upon clinical results, and the answer has consequently been but a vague generalisation, such as "I can cure some cases entirely, I can relieve others, but there are a great many cases that I cannot cure at all." One would wish to be able to give a more detailed answer than this, and if possible explain why some cases are cured more readily than others; so that, for any given patient under one's own care, one could form a definite idea beforehand as to how much benefit would accrue from the internal medication to be employed. We owe it to recent bacteriological work that we can do so.

### The Drugs.

The researches have been carried out both in animals and in man. The drugs used have been mainly three—salol, methylene blue, and urotropine. These have been given when there has been no bacteriuria, the inhibitive action of the urine being tested upon cultures of various micro-organisms both before and after the administration of the medicine; they have also been given when there have been various dgrees and kinds of bacteriuria, either pathological or experimental, and the results have been tested by making comparative cultivations from the urines before and after the administrations. The results have been put together, and they have pointed clearly to certain conclusions.

### Urinary Antiseptics.

In the first place, the fact that each of these drugs causes the urine to be less easily infected with micro-organisms than is ordinary urine has been fully confirmed. The administration of urotropine, methylene blue, and salol renders the urine of the patient so treated inhibitive to the growth of *Staphylococcus pyogenes*, *Streptococcus pyogenes*, *Bacillus typhosus*, *Bacillus coli communis*, and *Bacillus proteus vulgaris*, which were the organisms experimented with.

### Salol and Methylene Blue.

In the next place, salol is very much less effective in this respect than are the other two. This is a point of great importance, for salol, though rapidly being displaced by urotropine, is still widely used as a urinary antiseptic. It is interesting to note that methylene blue is fairly potent in inhibiting the growth of bacteria in urine, although it is not very widely used for this purpose, and perhaps it should be resorted to more often. It is not quite so effective as urotropine, but much more so than salol. There are some patients who cannot take urotropine continuously, and methylene blue is a useful alternative provided the patient is warned that his urine will be deep blue after it. The blue colour of the urine will persist for two days after a single dose of 2 grains, and it affords a very useful indication of the fact that the drug is being taken and absorbed. Urotropine and salol are usually prescribed in tablet form, and it is well-known that these tablets sometimes pass unchanged with the fæces; the urine itself will not spontaneously indicate whether or not the urotropine or the salol have been absorbed, as in the case of methylene blue, so that special tests have to be resorted to.

### Special Reacts.

*The Reactions for Urotropine in Urine.*—Urotropine by the mouth is excreted as formalin in the urine—hence its antiseptic action. The urine therefore reduces Fehling's solution, so much so as even to be mistaken for evidence of sugar. Potassium hydrate and resorcin give a red colour, which is fairly characteristic, and a similar but better red colour is given when caustic soda and phloroglucin are added to the urine—Jörgensen's test.

*The Reaction for Salol in Urine.*—Salol by the mouth is excreted as a phenyl compound, which, like other phenol bodies, gives a characteristic dark purple colour upon the addition of ferric chloride solution to the urine.

### Their Values.

In the third place *the above drugs cannot be given medicinally in sufficient quantity to make the urine actually bactericidal.* This is a very important point indeed; upon it depends the great limitation there is to the cure of many cases of bacteriuria by them. The antiseptic excretory products will *inhibit the growth* of the bacteria, but will not absolutely stop this growth, still less destroy the micro-organisms present. They render the urine an uncongenial medium for growth, but not an environment necessitating death. The effect of the drugs is weakest in the case of *Staphylococcus pyogenes*, and strongest in the case of *Bacillus typhosus* and *Streptococcus pyogenes*.

### Degrees of Bacteriuria.

Now bacteriuria may be of three degrees, as follows:—

I. The urine may contain bacteria which are being excreted by the urinary organs without these being themselves diseased. The bacilluria of enteric fever is a good example of this.

II. The urine may contain bacteria which are multiplying in the urinary passages, but which have not yet caused an actual breach of surface in the epithelial walls. Such is the case in the earlier stages of cystitis.

III. The urine may contain bacteria which are not only multiplying in the urine itself, but are also growing in the walls of the bladder or other part of the urinary tracts, with resultant catarrh or ulceration.

Seeing that the drugs merely check the rate of multiplication of bacteria in the urine itself it is particularly in the two first groups that their beneficial effects are marked; the urine is constantly being voided, carrying with it a certain number of micro-organisms every time, and if the rate of evacuation of organisms in this way exceeds their rate of multiplication a time comes when no organisms are left, and the condition is cured. When, however, there is multiplication of organisms in the walls of the bladder or kidney, the curative effects of the drugs are very much restricted; the drugs will inhibit the growth of the organisms already in the urine, and thereby the chance of re-infection of the wall is lessened; but the cure of the lesions in the walls themselves depends either upon the local application of stronger solutions of antiseptics by lavage, or by that greatest help of all, the "vis medicatrix naturæ": the drugs will inhibit re-infection, and the walls may then cure themselves.

### Indications for Treatment.

It is obvious, therefore, that urotropine, methylene blue, salol, or other similar drug should not be withheld until there is a definite lesion such as cystitis; when the latter has developed the best time for giving the drugs has gone by. They should be administered as soon as there is the least reason to suppose that organisms are likely to infect the urine. In cases of paraplegia, for example, they should be given before the pyuria appears, to prevent its coming on; when it has come on it is good to give the drugs, but it is better to have anticipated and prevented the bacteriuria. The same applies to all conditions when instruments are to be passed into the bladder—catheters, cystoscopes, lithotrity instruments, segregators, or the like; whenever possible a preliminary course of urotropine should be gone through, for no one can absolutely guarantee not to infect a bladder when an instrument is passed, especially if the passage has to be repeated often; the prophylaxis will greatly diminish the risk of any of the few organisms that may get in multiplying afterwards.

### Conclusion.

The main points which have been clearly proved by bacteriologists and clinicians together are therefore:—

I. That internal medication is most certainly of value, for it is possible to increase the anti-bacterial power of the urine thereby.

II. That such internal medication does not lead to the actual killing of the bacteria, but merely to the inhibition of their rate of growth. Cure therefore results much better when the bacteria are multiplying in the urine only than when they have gained a foothold in the walls of the urinary passages themselves. In incipient bacteriuria without cystitis success from internal medication is the rule and failure the exception; in long-standing bacteriuria, with cystitis, the reverse is true.

III. In all cases of bacteriuria internal medication is advisable whether other measures are used at the same time or not; inhibition of the growth of bacteria in the urine will diminish the chances of re-infection of the walls, and the latter, though not directly cured by the drugs, will stand a better chance of recovering by themselves.

IV. Internal medication is a potent means of prophylaxis against bacteriuria, and as such should be resorted to earlier than it sometimes is; that is to say, it should be used in prevention of pyuria in conditions under which pyuria is apt otherwise to occur.

The drugs act essentially by preventing bacteria from growing; and therefore accomplish more by way of prevention than by way of cure.

